# Rabbit Immune Cell Function: In Vitro Assays for Immunological Assessment Studies Using Flow Cytometry

**DOI:** 10.3390/biotech15020039

**Published:** 2026-05-29

**Authors:** Tamiris C. Sardinha, Philipe P. L. Pereira, Tamires A. R. Gomes, Bruna de A. C. F. Mendes, Stefani M. Ferreira, Leonila E. R. Raspantini, Cristina de O. Massoco, André T. Gotardo

**Affiliations:** Research Centre for Veterinary Toxicology (CEPTOX), Department of Veterinary Pathology, School of Veterinary Medicine and Animal Science, University of Sao Paulo, Pirassununga 13635-900, SP, Brazil; tamiris.sardinha@usp.br (T.C.S.); philipe.paschoal@usp.br (P.P.L.P.); tamiresassis.g@gmail.com (T.A.R.G.); bruna.abreucfm@usp.br (B.d.A.C.F.M.); esterraspantini@usp.br (L.E.R.R.); cmassoco@gmail.com (C.d.O.M.)

**Keywords:** lagomorph species, oxidative burst, phagocytosis, lymphocyte proliferation, immunotoxicology

## Abstract

Repeated blood collection in long-term studies in rodents is challenging and may compromise animal welfare. Rabbits represent a robust alternative model for longitudinal studies due to their larger blood volume and ease of repeated sampling. However, standardized assays to assess rabbit immune function remain scarce. This study presents standardized and optimized flow cytometry–based protocols for evaluating oxidative burst, phagocytosis, and lymphocyte proliferation in rabbits. Oxidative burst and phagocytic activity were analyzed in heparinized whole blood using DCFH and fluorescently labeled *Staphylococcus aureus*. Lymphocyte proliferation was assessed in CFSE-labeled PBMC stimulated with ConA. Flow cytometric analysis enabled simultaneous quantification of reactive oxygen species (ROS) generation, phagocytic uptake, and CFSE dilution modeling. Following stimulation with *S. aureus*, rabbit heterophils exhibited ROS production in a median of 90.4% (IQR: 84.8–92.9%) of gated cells, with a median phagocytic uptake of 23.6% (IQR: 13.4–27.2%). PMA-stimulated cells showed near-complete oxidative burst (median 99.1%, IQR: 98.7–99.5%), confirming their functional similarity to mammalian neutrophils. Lymphocytes exhibited measurable proliferative responses to ConA, validating PBMC-based assays for adaptive immune assessment. These standardized methods offer a framework for investigating innate and adaptive immune functions in rabbits, contributing to immunotoxicological and safety evaluation studies.

## 1. Introduction

In long-term efficacy, safety and toxicology studies in rodents, repeated blood collection can be challenging and compromise animal welfare. It is often difficult to obtain sufficient sample volumes without disrupting the animal’s homeostasis [1,2,3]. To reduce animal use, rabbits are suggested as suitable models for repeated blood sampling without requiring euthanasia, allowing collection at multiple time points, unlike in rodents, without causing serious adverse effects [4,5,6]. However, most existing protocols and assays developed for assessing polymorphonuclear (PMN) cell functions, including reactive oxygen species (ROS) generation and phagocytic activity, were originally designed for mice, humans, or other species. Since ROS production by circulating PMNs upon activation is a crucial mechanism in microbial killing and phagocytosis, it provides essential insights into innate immune function and host defense mechanisms [7,8,9,10].

Briefly, protocols using 2′,7′-dichlorofluorescein diacetate (DCFH) allow fluorescent detection of intracellular ROS through oxidization of non-fluorescent DCFH to 2′,7′-dichlorofluorescein (DCF). The resulting fluorescence intensity is directly proportional to the cellular oxidative activity. This approach is widely used to assess the oxidative burst capacity of neutrophils and monocytes upon stimulation with agents such as phorbol 12-myristate 13-acetate (PMA) or during phagocytosis [11,12].

Phagocytosis assays are performed by incubating freshly isolated leukocytes with heat-killed, fluorescently labeled *Staphylococcus aureus*, *Escherichia coli*, or Zymosan particles tagged with propidium iodide or fluorescein isothiocyanate (FITC). Phagocytic activity is quantified by flow cytometry based on the percentage of fluorescence-positive cells and the mean fluorescence intensity (MFI) of internalized particles [13,14,15].

In vitro culture of isolated peripheral blood mononuclear cells (PBMCs) allows the assessment of lymphocyte proliferative capacity in individual animals following stimulation with different mitogens. Established protocols using 5(6)-carboxyfluorescein diacetate N-succinimidyl ester (CFSE) enable the tracking of lymphocyte divisions, as the dye is equally partitioned between daughter cells. Consequently, each successive generation exhibits approximately half the fluorescence intensity of its parent population [16,17,18].

Despite the widespread use of rabbits in toxicology and safety testing, standardized protocols for the immunological assessment in this species remain limited, particularly regarding cellular immune functions in toxicological and immunotoxicological studies [19]. Establishing reliable in vitro functional assays is essential for identifying potential immunomodulatory effects of tested compounds and monitoring immune homeostasis over time. This study establishes a refined framework for evaluating lymphoproliferative responses, oxidative burst, and phagocytic capacity in peripheral blood heterophils and mononuclear cells from rabbits. These approaches provide a framework for comprehensive evaluation of both innate and adaptive immune functions using flow cytometry-based functional assays, thereby contributing to the refinement of immunotoxicological studies in this animal model. To the best of our knowledge, no previous studies have attempted to analyze rabbit leukocyte immune function using flow cytometry.

## 2. Materials and Methods

The experimental procedures were approved by the Ethics Committee on the Use of Animals (CEUA) of the School of Veterinary Medicine and Animal Science, University of São Paulo (FMVZ-USP; protocol code CEUA 8425111022). The study was conducted at the University of São Paulo (USP), “Fernando Costa” campus, located in Pirassununga, São Paulo, Brazil (21°59′46.00″ S, 47°25′32.99″ W). All animal care and handling were performed by trained personnel under veterinary supervision, in accordance with institutional ethical guidelines.

### 2.1. Animal Housing and Feeding

Twenty adult male New Zealand White rabbits (*Oryctolagus cuniculus*), six months of age, with a mean body weight of 4.0 ± 0.1 kg, were used in this study. The animals were housed individually in suspended wire cages (80 × 60 × 40 cm) under controlled environmental conditions, including a temperature of 22 ± 3 °C, relative humidity of 45–65%, and a 12 h light/dark cycle. Rabbits were fed a species-specific commercial diet (Coelhão^®^, Guabi Nutrição e Saúde Animal Ltda., Campinas, SP, Brazil) at 120 g per animal per day, and water was provided ad libitum through automatic drinking systems.

### 2.2. Immunotoxicological Assays

#### 2.2.1. Determination of Oxidative Burst and Phagocytic Activity

Oxidative burst and phagocytic activity were evaluated by flow cytometry using heparinized whole blood from all 20 animals [14]. Briefly, 100 µL samples were divided into two cytometry tubes containing phosphate-buffered saline (PBS, q.s. 1 mL) and subjected to two experimental conditions: (i) DCFH (250 µM) with *S. aureus* (pHrodo™ Red, Thermo Fisher Scientific, Waltham, MA, USA) (Invitrogen A10010, 100 µg/mL), or (ii) DCFH (250 µM) with PMA (phorbol myristate acetate; 1 µg/mL).

Samples were incubated at 37 °C for 30 min under gentle agitation and protected from light. The reaction was stopped by adding 2 mL of ice-cold 3 mM EDTA to prevent cell aggregation and stop phagocytosis. After centrifugation (450× *g*, 5 min, 4 °C), the cell pellet underwent RBC lysis with chilled ACK buffer (2 mL, 10 min, 2–4 °C), a step repeated until complete lysis was achieved. The remaining leukocytes were resuspended in 200 µL of PBS for flow cytometric analysis.

Control tubes were included to ensure proper fluorescence compensation and cell integrity assessment, including blank (PBS only), single-stain DCFH-DA, and single-stain *S. aureus* only. This configuration enabled simultaneous evaluation of phagocytic capacity and intracellular ROS production in peripheral PMN cells while facilitating accurate compensation for spectral overlap between fluorescent channels.

Species-specific adaptations for rabbit samples included the use of logarithmic scaling for forward scatter (FSC) and side scatter (SSC) parameters, which provided superior resolution for discriminating polymorphonucleated populations from residual erythrocytes following ACK lysis. While unconventional for some cell types, logarithmic scaling for FSC and SSC parameters was used, as it improved discrimination of rabbit heterophils compared to linear scaling. This gating approach was uniformly applied across all 20 animals. Additionally, since commercially validated anti-rabbit leukocyte surface markers (e.g., CD45) are scarcely available, cell populations were identified exclusively by scatter properties. The limitations of this approach are discussed below.

#### 2.2.2. Lymphocyte Proliferation Assay

The lymphocyte proliferation assay was performed as previously described [20,21], with minor adaptations for rabbits. PBMCs were isolated by density gradient centrifugation using 3 mL of whole blood diluted 1:1 PBS and layered over 3 mL of Ficoll-Paque^®^ Plus (Cytiva, Marlborough, MA, USA). After isolation, cells were resuspended in RPMI-1640 supplemented with 10% fetal bovine serum (FBS) and 1% gentamicin, incubated for 10 min at room temperature, and centrifuged (270× *g*, 7 min, 4 °C). The resulting cell pellet was washed with PBS prior to labeling.

For CFSE staining, cells were resuspended in 1 mL of PBS and incubated with 5 µM CFSE-DA for 10 min at 37 °C on a rocking rotator, protected from light. The reaction was stopped with 2 mL of heat-inactivated FBS, followed by centrifugation (350× *g*, 5 min, 4–8 °C). The resulting pellet exhibited a characteristic lime-green color, indicating effective labeling.

Lymphocytes were cultured in 96-well U-bottom plates, with 2 × 10^5^ cells per well in 100 µL, reaching a final volume of 200 µL after adding the respective stimuli. The culture medium was RPMI-1640 supplemented with 10% FBS, 2 mM L-glutamine, 10 mM HEPES, 100 U/mL penicillin, 100 µg/mL streptomycin, and 50 µM 2-mercaptoethanol.

Two conditions were evaluated in triplicate: (i) negative control (no stimulus), (ii) concanavalin A (ConA; 5 µg/mL), which served as a specific positive stimulus for lymphocyte proliferation. Plates were incubated for 120 h at 37 °C in a humidified atmosphere with 5% CO_2_. After incubation, triplicate wells were pooled, centrifuged (270× *g*, 7 min, 4 °C), and resuspended in 150 µL PBS for flow cytometry analysis.

Flow cytometry was performed on a BD FacsCalibur™ (Becton Dickinson Immunocytometry Systems^®^, San Jose, CA, USA) using FL-1 (530 nm ± 15 nm) for DCFH (Sigma-Aldrich^®^ Brasil Ltda., Barueri, SP, Brazil #D6883) and CFSE (Sigma-Aldrich^®^-#21888), also filter FL-2 (585 nm ± 21 nm) for *S. aureus* pHrodo™ Red. Oxidative burst and phagocytosis were analyzed immediately following staining. Data were processed using FlowJo™ v10.10.0 (BD Life Sciences, Ashland, OR, USA).

### 2.3. Statistical Analysis

Data are presented descriptively. Continuous variables are expressed as median and interquartile range (IQR), given the small sample size and the non-parametric nature of the distributions. All flow cytometric analyses were performed in FlowJo™ v10.10.0 (BD Life Sciences, Ashland, OR, USA). Derived metrics include: percentage of DCFH-positive cells (Q2 + Q3 quadrants, reflecting total oxidative burst activity), percentage of SA-positive cells (Q1 + Q2 quadrants, reflecting total phagocytic uptake), proliferative index (PI), division index (DI), and percentage of divided cells (PDC), as computed by the FlowJo™ Proliferation Modeling tool.

## 3. Results

### 3.1. Gating Strategy and Leukocyte Discrimination

**Figure 1:**
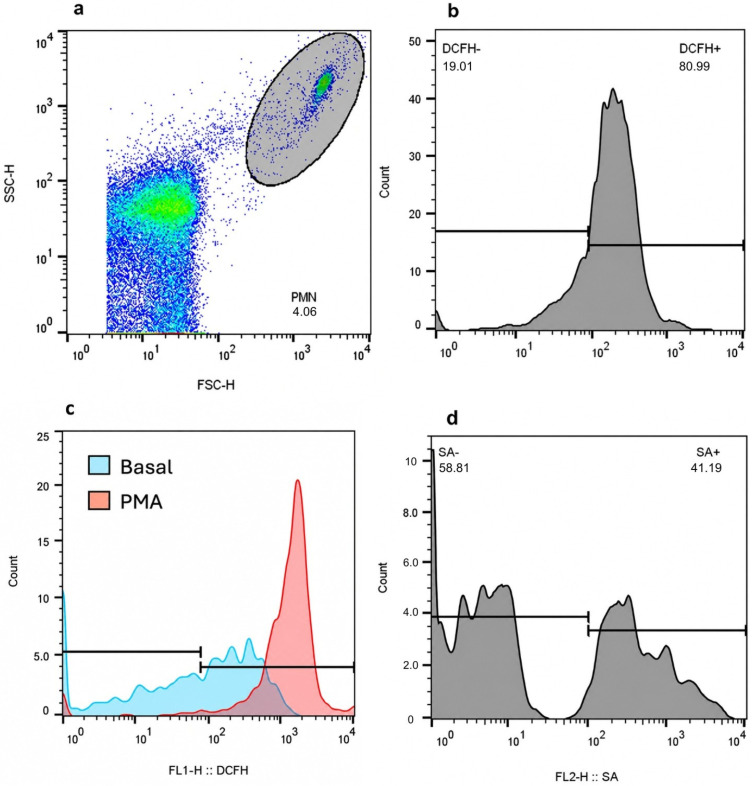
Representative dot plots and histograms illustrating the gating strategy for oxidative burst and phagocytosis assays in rabbit peripheral blood. (**a**) Dot plot of forward scatter (FSC, log scale) versus side scatter (SSC, log scale) showing the gated polymorphonucleated population, selected to exclude debris and residual erythrocytes remaining after ACK lysis. (**b**) Histogram of FL-1 fluorescence (DCFH; 530 nm) from the gated leukocyte population in the unstimulated or baseline condition, demonstrating basal intracellular ROS levels. (**c**) Histogram of FL-1 fluorescence (DCFH) from the same gated population following PMA stimulation (1 µg/mL), showing a rightward shift in fluorescence intensity consistent with acti-vation of oxidative burst. (**d**) Histogram of FL-2 fluorescence (*S. aureus* pHrodo™ Red; 585 nm) from the gated PMN population in the *S. aureus* stimulated condition, showing a shift in fluo-rescence intensity consistent with internalization of fluorescently labeled bacterial particles by heterophils.

Rabbit erythrocytes are particularly sensitive to ACK lysis buffer, enabling selective lysis within minutes while preserving leukocyte integrity. However, residual intact erythrocytes persisted during flow cytometric analysis. A logarithmic scale for forward (FSC) and side (SSC) scatter provided superior resolution for discriminating leukocyte populations (Figure 1a).

The gated population on dot plot of Figure 1a represents a relatively homogeneous cluster defined by cell size (FSC) and internal complexity (SSC) and was considered for functional analysis to exclude all debris from the gate of analysis. When gating this cell population of larger size and higher granularity, DCFH-stained leukocytes, exhibited increased fluorescence intensity in the FL-1 channel (Figure 1b). Fluorescence intensity further increased upon PMA stimulation, confirming activation of the oxidative burst response (Figure 1c). For phagocytosis analysis, a similar gating strategy was performed, with FL-2 distinguishing heterophils that had phagocytosed labeled *S. aureus* (Figure 1d).

**Figure 2:**
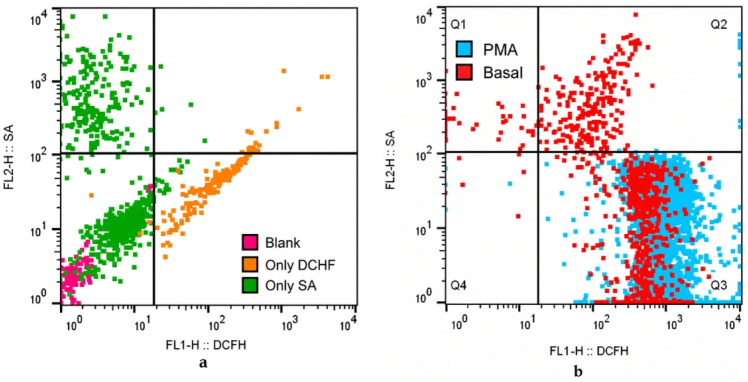
Fluorescence compensation controls and quadrant analysis of oxidative burst and phagocytic activity in rabbit peripheral blood heterophils. (**a**) Single-stain controls (blank, pink; DCFH-only, orange; *S. aureus* pHrodo™ Red-only, green) confirming absence of spectral spillover between FL1 and FL2 channels and used to define positivity thresholds and quadrant gates. (**b**) Representative dot plot of gated PMN cells from *S. aureus*-stimulated (DSA; basal, red) and PMA-stimulated (blue) conditions. Quadrant gates (defined by vertical and horizontal lines) separate cell populations based on DCFH fluorescence (ROS production, FL1) and *S. aureus* uptake (FL2). Q1: DCFH−/SA+; Q2: DCFH+/SA+; Q3: DCFH+/SA−; Q4: DCFH−/SA−.

To validate fluorescence compensation and confirm the specificity of each staining component, single-stain control samples were analyzed prior to experimental acquisitions. Figure 2b illustrates representative dot plots comparing heterophils from the *S. aureus*-stimulated condition (DSA; basal, red) and PMA-stimulated condition (blue). In the DSA condition, cells distributed across both DCFH-positive and SA-positive quadrants, reflecting simultaneous ROS production and phagocytic uptake of bacterial particles. In contrast, PMA-stimulated cells accumulated predominantly in Q3 (DCFH+, SA−), consistent with maximal oxidative burst activation in the absence of bacterial particles.

### 3.2. Oxidative Burst and Phagocytic Activity

Quantitative analysis of oxidative burst and phagocytic activity across all 20 animals is summarized in Table 1. Following stimulation with *S. aureus* pHrodo™ (DSA), rabbit heterophils demonstrated strong ROS production, with a median of 90.4% (IQR: 84.8–92.9%) of gated PMNs classified as DCFH-positive (Q2 + Q3; Figure 2b). Phagocytic uptake (SA-positive cells, Q1 + Q2) was detected in a median of 23.6% (IQR: 13.4–27.2%) of PMNs. In the PMA-stimulated group (positive control for oxidative burst), near-complete activation was observed, with a median of 99.1% (IQR: 98.7–99.5%) DCFH-positive cells and negligible SA-positive fractions (median 0.2%), as expected since PMA does not involve bacterial particles (Figure 2b). Unstimulated blank samples showed minimal background fluorescence (DCFH+: median 3.5%; SA+: median 0.1%), consistent with the compensation controls shown in Figure 2a. These results confirm the functionality of rabbit heterophils and the robustness of the assay controls.

### 3.3. Lymphocyte Proliferation

**Figure 3:**
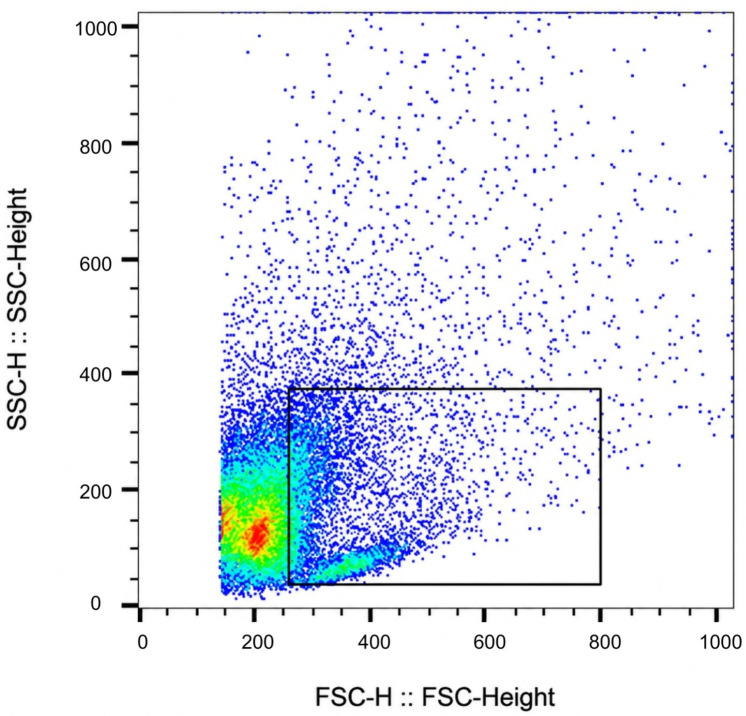
Gating strategy for lymphocyte proliferation analysis by CFSE dilution. Representative dot plot illustrating the scatter-based gating of lymphocytes from rabbit PBMC cultures. The gate was defined using an SSC threshold of approximately 400 to exclude polymorphonuclear cells (heterophils), and an FSC range between 200 and 800 to include proliferating lymphocytes while excluding small necrotic or debris events falling below the lower FSC threshold.

For the lymphoproliferation assay, cells recovered from culture plates were transferred to tubes for cytometry analysis. Data were analyzed in FlowJo™ v.10.10.0 using the “Proliferation Modeling” tool, which generates CFSE fluorescence histograms (FL1) from a gated lymphocyte population. Gating was defined using an SSC threshold of approximately 400 to exclude PMNs, and an FSC range between 200 and 800 to include proliferating lymphocytes while excluding small necrotic cells below this threshold (Figure 3).

**Figure 4:**
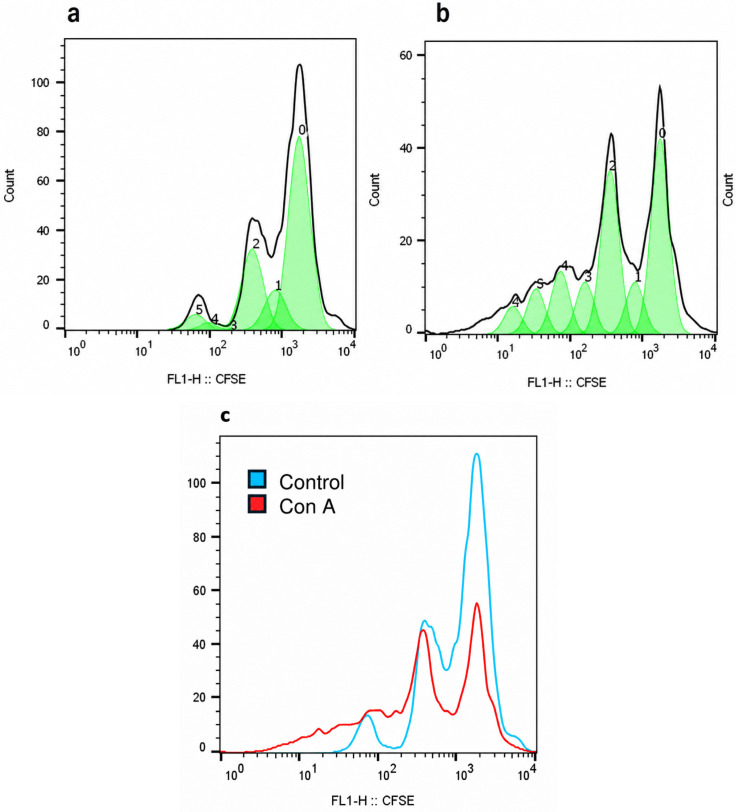
Proliferation modeling of cultured rabbit lymphocytes stimulated with ConA. (**a**) CFSE fluorescence profile of unstimulated lymphocytes (negative control), showing minimal proliferative activity. (**b**) CFSE fluorescence profile of ConA-stimulated lymphocytes showing distinct proliferation peaks across multiple generations. (**c**) Overlay of unstimulated control (blue line) and ConA-stimulated lymphocytes (red line). In panels (**a**,**b**), the black line represents the overall CFSE fluorescence distribution, and the green peaks represent distinct cell generations identified by the FlowJo™ Proliferation Modeling algorithm based on successive CFSE fluo-rescence dilution. Each peak corresponds to a cell division cycle, with the rightmost peak (highest fluorescence intensity) representing the undivided parental population (generation 0). The number of peaks identified reflects the number of completed cell division rounds detected within the 120-h culture period.

Peaks corresponding to successive cell divisions were automatically identified by the software, allowing calculation of the proliferation index (PI), division index (DI), and percentage of divided cells (PDC). To illustrate the applicability of this approach in rabbit lymphocytes, a representative example is shown in Figure 4, demonstrating the characteristic CFSE dilution pattern obtained following ConA stimulation. The ConA-stimulated culture displayed multiple successive fluorescence dilution peaks, consistent with active proliferation, whereas the unstimulated control showed a predominant undivided population. This representative example demonstrates that the FlowJo Proliferation Modeling algorithm could resolve individual cell generations from rabbit lymphocyte cultures, providing quantitative proliferation metrics (PI, DI, and PDC) as output for each sample.

## 4. Discussion

Rabbits exhibit distinctive hematological characteristics compared with many mammalian species, particularly in their leukocyte distribution, where circulating lymphocytes predominate over granulocytes [22]. Despite their lower abundance, rabbit heterophils demonstrated functional activity in the present study, showing oxidative burst and phagocytic capacity following *S. aureus* stimulation. These findings are consistent with previous reports describing rabbit granulocyte antimicrobial responses and support the concept that heterophils functionally resemble mammalian neutrophils despite species-specific morphological differences [23,24]. The high percentage of DCFH-positive cells observed after bacterial stimulation (median 90.4%) and PMA activation (median 99.1%) suggests preserved activation of NADPH oxidase–dependent ROS generation pathways, which are central to innate immune defense and microbial killing. Comparable oxidative burst responses have been reported in activated human and murine neutrophils, reinforcing the notion of conserved effector mechanisms across vertebrate species despite differences in leukocyte composition and nomenclature [25].

The lower proportion of phagocytic-positive cells relative to oxidative burst activity may reflect differences in the temporal dynamics between particle internalization and ROS generation, a phenomenon previously described in flow cytometric studies of equine and murine granulocytes [13,26,27] Importantly, the successful recovery of viable PBMCs and detection of ConA-induced lymphocyte proliferation demonstrates that rabbit peripheral blood can support complementary evaluation of adaptive immune responses. Together, these findings strengthen the value of rabbits as an experimental model for immunotoxicological investigations, particularly in longitudinal studies requiring repeated blood sampling [6,28,29].

### Limitations

Several limitations of the present study should be acknowledged. First, PMN cell populations were identified exclusively based on FSC and SSC scatter parameters using logarithmic scaling, without the aid of immunophenotypic surface markers (e.g., CD45) or nuclear dyes (e.g., SYTO). This approach, necessitated by the limited commercial availability of validated anti-rabbit leukocyte antibodies, prevents definitive confirmation of gate purity and cannot exclude minor contamination by monocytes, which also exhibit phagocytic and oxidative burst activity. Future studies should consider incorporating a pan-leukocyte marker to improve gating stringency. Second, the use of logarithmic FSC/SSC scaling resulted in a proportion of events near the chart edges (18–22% depending on sample), which may have caused some heterophils to be excluded from analysis. Third, a live/dead discrimination dye (e.g., fixable viability dye) was not used in the oxidative burst and phagocytosis assays, nor in the 120-h lymphocyte proliferation cultures. Dead cells and debris may non-specifically bind DCFH or CFSE and contribute to autofluorescence. The inclusion of a viability marker in future protocols is strongly recommended, particularly for long-term culture-based assays. Fourth, the absence of a direct comparison with another species assessed under identical experimental conditions limits the strength of the cross-species functional claim. The comparison presented in the introduction is based on the available literature and is subject to inter-laboratory and inter-instrument variability.

## 5. Conclusions

In this study, we propose optimized flow cytometry–based protocols and analysis workflows for evaluating functional immune responses in rabbits, thereby filling an important gap in immunotoxicological assessment. Rabbit heterophils demonstrated strong and consistent oxidative burst and phagocytic responses, with quantitative data validated across 18 animals per condition, after exclusion of samples that did not meet quality criteria (*n* = 18). Lymphocyte proliferation assays provided measurable CFSE dilution profiles following ConA stimulation. Further studies incorporating cytokine profiling and surface marker characterization could complement these functional assays, enabling a more comprehensive understanding of rabbit immune homeostasis under toxicological or immunomodulatory conditions.

## Figures and Tables

**Table 1:** Descriptive statistics of oxidative burst (DCFH+) and phagocytic activity (SA+) in rabbit peripheral blood PMNs across experimental conditions. Values expressed as median (interquartile range). DCFH+ = Q2 + Q3 quadrants; SA+ = Q1 + Q2 quadrants.

Group/Parameter	N	DCFH+ % (Burst)	SA+ % (Phagocytosis)
Blank (unstimulated)	2	3.5% (2.1–5.0%)	0.1% (0.1–0.2%)
SA (*S. aureus* stimulus)	18	90.4% (84.8–92.9%)	23.6% (13.4–27.2%)
PMA (positive control)	18	99.1% (98.7–99.5%)	0.2% (0.0–0.2%)

## Data Availability

The original contributions presented in this study are included in the article. Further inquiries can be directed to the corresponding author.

## Abbreviations

The following abbreviations are used in this manuscript:

ACK	Ammonium-Chloride-Potassium
CFSE	5(6)-carboxyfluorescein diacetate N-succinimidyl ester
DCF	2′,7′-dichlorofluorescein
DCFH	2′,7′-dichlorofluorescein diacetate
EDTA	Ethylenediaminetetraacetic Acid
FBS	Fetal Bovine Serum
FITC	Fluorescein Isothiocyanate
FSC	Forward Scatter
HEPES	N-2-hydroxyethylpiperazine-N-2-ethane sulfonic acid
MFI	Mean Fluorescence Intensity
PMA	phorbol 12-myristate 13-acetate
PMN	Polymorphonuclear
PBMCs	Peripheral Blood Mononuclear Cells
PBS	Phosphate-buffered Saline
RBC	Red Blood Cell
ROS	Reactive Oxygen Species
SSC	Side Scatter

## References

[1] Chapman K., Burnett J., Corvaro M., Mitchell D., Robinson S., Sangster T., Sparrow S., Spooner N., Wilson A. (2014). Reducing pre-clinical blood volumes for toxicokinetics: Toxicologists, pathologists and bioanalysts unite. Bioanalysis.

[2] Sewell F., Corvaro M., Andrus A., Burke J., Daston G., Delaney B., Domoradzki J., Forlini C., Green M.L., Hofmann T. (2022). Recommendations on dose level selection for repeat dose toxicity studies. Arch. Toxicol..

[3] Li X., Li X., Wu Z., Wang K., Qiao R., Han X., Li X., Yang F., Yu T., Wang T. (2025). Recent advances in nutritional requirements and metabolic homeostasis regulation of animals under stress conditions. Animals.

[4] Foote R.H., Carney E.W. (2000). The rabbit as a model for reproductive and developmental toxicity studies. Reprod. Toxicol..

[5] Parasuraman S., Raveendran R., Kesavan R. (2010). Blood sample collection in small laboratory animals. J. Pharmacol. Pharmacother..

[6] Ferreira S.M., Zapparoli H., Mussi C.S., Maiorka P.C., Andrade A.F.C., Górniak S.L., Gotardo A.T. (2026). Systemic toxicity of L-mimosine in rabbits: A non-rodent model for safety assessment. J. Appl. Toxicol..

[7] Mittal M., Siddiqui M.R., Tran K., Reddy S.P., Malik A.B. (2014). Reactive Oxygen Species in Inflammation and Tissue Injury. Antioxid. Redox Signal..

[8] Nguyen G.T., Green E.R., Mecsas J. (2017). Neutrophils to the ROScue: Mechanisms of NADPH Oxidase Activation and Bacterial Resistance. Front. Cell. Infect. Microbiol..

[9] Liu J., Han X., Zhang T., Tian K., Li Z., Luo F. (2023). Reactive oxygen species (ROS) scavenging biomaterials for anti-inflammatory diseases: From mechanism to therapy. J. Hematol. Oncol..

[10] Tocu G., Ștefănescu B.I., Matei L.S., Țocu L. (2025). Phagocyte NADPH oxidase NOX2-derived reactive oxygen species in antimicrobial defense: Mechanisms, regulation, and therapeutic potential—A narrative review. Antioxidants.

[11] De Haan L.R., Reiniers M.J., Reeskamp L.F., Belkouz A., Ao L., Cheng S., Ding B., van Golen R.F., Heger M. (2022). Experimental Conditions That Influence the Utility of 2’7’-Dichlorodihydrofluorescein Diacetate (DCFH2-DA) as a Fluorogenic Biosensor for Mitochondrial Redox Status. Antioxidants.

[12] Walrand S., Valeix S., Rodriguez C., Ligot P., Chassagne J., Vasson M.-P. (2003). Flow cytometry study of polymorphonuclear neutrophil oxidative burst: A comparison of three fluorescent probes. Clin. Chim. Acta.

[13] Flaminio M.J.B.F., Rush B.R., Davis E.G., Hennessy K., Shuman W., Wilkerson M.J. (2002). Simultaneous flow cytometric analysis of phagocytosis and oxidative burst activity in equine leukocytes. Vet. Res. Commun..

[14] Hasui M., Hirabayashi Y., Kobayashi Y. (1989). Simultaneous measurement by flow cytometry of phagocytosis and hydrogen peroxide production of neutrophils in whole blood. J. Immunol. Methods.

[15] Wojcicka-Lorenowicz K., Kostro K., Lisiecka U., Gasiorek B. (2018). Phagocytic activity and oxygen metabolism of peripheral blood granulocytes from rabbits experimentally infected with Trichophyton mentagrophytes. J. Vet. Res..

[16] Bocharov G., Luzyanina T., Cupovic J., Ludewig B. (2013). Asymmetry of Cell Division in CFSE-Based Lymphocyte Proliferation Analysis. Front. Immunol..

[17] Weston S.A., Parish C.R. (1990). New fluorescent dyes for lymphocyte migration studies. J. Immunol. Methods.

[18] Wei S., Rosen H., Matheu M., Sanna M.G., Wang S.-K., Jo E., Wong C.-H., Parker I., Cahalan M.D. (2005). Sphingosine 1-phosphate type 1 receptor agonism inhibits transendothelial migration of medullary T cells to lymphatic sinuses. Nat. Immunol..

[19] Sokolowski K., Turner P.V., Lewis E., Wange R.L., Fortin M.C. (2024). Exploring rabbit as a nonrodent species for general toxicology studies. Toxicol. Sci..

[20] Dobrovolskaia M.A., McNeil S.E. (2013). Understanding the correlation between in vitro and in vivo immunotoxicity tests for nanomedicines. J. Control. Release.

[21] Roy K., Shokhirev M.N., Mitchell S., Hoffmann A. (2018). Deriving Quantitative Cell Biological Information from Dye-Dilution Lymphocyte Proliferation Experiments.

[22] Siegel A., Walton R.M., Quesenberry K.E., Carpenter J.W. (2020). Hematology and biochemistry of small mammals. Ferrets, Rabbits, and Rodents: Clinical Medicine and Surgery.

[23] Cohn Z.A., Morse S.I. (1959). Interactions between rabbit polymorphonuclear leukcocytes and Staphylococci. J. Exp. Med..

[24] Freischlag J., Backstrom B., Kelly D., Keehn G., Busuttil R.W. (1986). Comparison of blood and peritoneal neutrophil activity in rabbits with and without peritonitis. J. Surg. Res..

[25] Esteves P.J., Abrantes J., Baldauf H.M., BenMohamed L., Chen Y., Christensen N., González-Gallego J., Giacani L., Hu J., Kaplan G. (2018). The wide utility of rabbits as models of human diseases. Exp. Mol. Med..

[26] Massoco C., Palermo-Neto J. (2003). Effects of midazolam on equine innate immune response: A flow cytometric study. Vet. Immunol. Immunopathol..

[27] Gardner R., Nydam D., Luna J., Bicalho M., Matychak M., Flaminio M. (2007). Serum opsonization capacity, phagocytosis, and oxidative burst activity in neonatal foals in the intensive care unit. J. Vet. Intern. Med..

[28] Christopher M.M., Hawkins M.G., Burton A.G. (2014). Poikilocytosis in rabbits: Prevalence, type, and association with disease. PLoS ONE.

[29] Genovese K.J., He H., Swaggerty C.L., Kogut M.H. (2013). The avian heterophil. Dev. Comp. Immunol..

